# The Attending of the Day (“The Pretending”): An Exercise in Autonomy

**DOI:** 10.7759/cureus.31134

**Published:** 2022-11-05

**Authors:** Regina Makdissi, Naren Nallapeta, Eric Moss, Archana Mishra, Roberto O Diaz Del Carpio

**Affiliations:** 1 Internal Medicine, University at Buffalo, Buffalo, USA; 2 Internal Medicine, State University of New York, Buffalo, USA; 3 Medicine, Utah Valley Hospital, Utah, USA; 4 Internal Medicine, CareMore Health, Cerritos, USA

**Keywords:** creative problem solving, medical resident education, learning environment, innovation in medical education, learner autonomy

## Abstract

Background

As regulations governing appropriate resident supervision increase, it has become increasingly difficult to provide residents with the appropriate level of autonomy during their training years. The “Attending of the Day” describes an experiential teaching method that provides a balance between learners’ autonomy and appropriate supervision.

Methodology

Each day one member of the inpatient team is selected as the “Attending of the Day,” or “The Pretending.” She or he then performs the typical duties of the teaching faculty, from medical decision-making regarding patient care to educating other team members during rounds. “The Pretending” is directly supervised by the clinical faculty.

Results

Using the grounded theory methodology, we analyzed 935 anonymous evaluations from students and residents over 14 years, leading to the identification of the following three major themes: created an enabling learning environment, provided autonomy, and improved confidence. These results led to the inclusion of the technique as part of the Back to Bedside initiative, which was rated as an essential tool in building confidence and autonomy by 75% of the participants in the 2018 Accreditation Council for Graduate Medical Education’s Back to Bedside residents’ well-being survey. Recently, the Jacobs School of Medicine launched the Moments of Excellence in Education: Recognition and Inspiration (MEE:RI) program which gives students a way to recognize exemplary moments of teaching they encounter. The “Attending of the Day” method received recognition as a transformative experience in students’ medical education.

Conclusions

The “Attending of the Day” is the first innovative experiential learning technique that allows learners of all levels in both Undergraduate Medical Education (UME) and Graduate Medical Education (GME) to practice and assess autonomy. This innovation suggests that residents and students are looking for opportunities to challenge themselves. “The Pretending” allows them to experience those challenges in an empowering learning environment while they gradually build their confidence on the path to achieving progressive autonomy.

## Introduction

Given the various changes in policies requiring more supervision, reducing work hours, and curtailing autonomy, it has become increasingly difficult to provide learners with the “appropriate level of autonomy” let alone assess residents’ readiness to practice independently [1]. The entrustment of clinical tasks to medical trainees is a seemingly easy process that occurs multiple times each day in nearly every clinical setting where medical students, residents, or fellows are trained. Yet, when analyzed, many factors appear to determine how, when, and whether learners are granted responsibilities under indirect or distant supervision [2].

Clinical supervision incorporates the “ability to anticipate a doctor’s strengths and weaknesses in particular clinical situations in order to maximize patient safety.” If supervision is excessive and more than necessary, trainees may not be able to progress toward competence and may in fact participate less actively in clinical decision-making. Conversely, inadequate supervision can compromise patient safety and trainees’ learning experiences [3-5].

Recent research on the role of trust in clinical supervision suggests five factors that influence trust: supervisor characteristics, trainee characteristics, the supervisor-trainee relationship, the context in which supervision occurs, and the task itself [6]. Ten Cate et al. analyzed the mechanisms that affect the entrustment of trainees to make decisions in graduate and undergraduate medical education [2]. Anticipating a decision to grant autonomy at a designated level of supervision appears to align better with healthcare practice than most current assessment practices.

Williams and Deci summarized the strong evidence supporting that autonomy-enabling medical educators facilitate more humanistic healthcare beliefs and behaviors and promote improved conceptual learning and psychological adjustment in their learners [7].

So, how do we achieve the appropriate balance between supervision and autonomy?

The aim of this paper is to describe an experiential learning model in which residents and students lead an entire team of patients and learners. It illustrates how providing supervised autonomy using the experiential learning theory helps both learners and teachers assess the learners’ readiness for autonomy. Therefore, faculty will be able to simultaneously provide trainees and students with supervision and autonomy and accurately evaluate learners’ progression in the milestones and entrustable professional activities (EPA).

The innovation has the following objectives: (1) create a supportive learning environment where all team members are comfortable contributing to the discussion; (2) enhance learners’ problem-solving skills; (3) empower learners to perform a critical self-assessment of their management, leadership, and knowledge skills.

## Materials and methods

The “Attending of the Day” technique had been used by the faculty author since 2008 on the inpatient teaching service. When preparing for the Back to Bedside initiative and during focus group discussions, this technique was identified as an intervention that would promote residents’ autonomy. The team analyzed the faculty evaluations pertaining to the “Attending of Day” in 2017 and included survey questions in our follow-up survey in 2018 to gauge the effectiveness of this experiential learning technique. Follow-up analysis of anonymous evaluations continued until 2022.

The “Attending of the Day” takes place on the inpatient medical floors for any teaching team size, and the learners are third and fourth-year medical students, interns, and team residents. An orientation to the innovation along with the expectations are provided at the beginning of the week with the teaching attending. The “Attending of the Day” has mainly been implemented on the hospitalist inpatient teaching services but could also be used on subspecialty rotations (while doing consults, for example). This could also be used for other specialty training programs, including family medicine, pediatrics, and surgical specialties.

Program description

Attending of the Day is the first described innovative experiential learning technique that allows internal medicine residents to practice autonomy regardless of the style of the supervisors (micro-managers or hands-off). This innovation confirmed that residents and students are always looking for opportunities to challenge themselves and this method creates an empowering learning environment to assess learners’ readiness and achieve progressive autonomy.

“Attending of the Day” provides trainees with an opportunity to practice and rehearse their autonomy, and supervisors to observe, evaluate, and provide feedback to the learners. It creates a safe and unintimidating culture to share one’s thought process, recognize limitations, and identify areas for improvement for each member of the inpatient team. As described in Table 1, the experience is both challenging and fun.

**Table 1:** A practical guide to implementing Attending of the Day.

A practical guide to implementing attending of the day
Pre-select patients in the team who are agreeable to take part in the exercise: this could take place during a new inpatient visit, follow-up visit, or even an encounter with family members
Explain the purpose of the exercise
Provide constructive feedback to the learner and allow patients to provide their feedback
At the beginning of the rotation, provide a description of each role including that of the attending
Start with residents in the team; this provides an opportunity for the junior members to observe and modify their techniques when it is their turn
When the Attending of the Day is not sure about the next step, open the discussion to the rest of the team members (ask them to vote); you get to vote last
During presentations and discussions, write down your thoughts and questions to ask at the end of each case
Ask the Attending of the Day for feedback about what worked well and what, if any, changes they would make in the future regarding their role
Provide constructive feedback

Description of the exercise

Every day one member of the inpatient team becomes the Attending of the Day, or “The Pretending.” The roles of the Attending of the Day (Table 2) and faculty (Table 3) are described below.

**Table 2:** Description of the Attending of the Day exercise. "The Pretending": The Attending of the Day

The responsibilities of The Pretending
Discuss the management of all patients with the team and propose final decisions regarding their care, pending approval of the faculty. If the faculty disagrees with the plan, they need to explain their thought process
If there are two approaches to the problem, the faculty might decide to proceed with attending of the day’s approach
Make clinical decisions across the wide spectrum, from repleting electrolytes, starting or stopping antibiotics, or when to discharge a patient
Seek the opinions and thought processes of other team members when uncertain of the next step
Assign topics of discussion to the other team members and provide didactic teaching at least once during the rotation
Perform bedside rounds on pre-selected patients who are aware of the exercise

**Table 3:** The responsibilities of the faculty.

The responsibilities of the faculty
Observe the Attending of the Day for management skills, on-the-spot thought process, clinical reasoning, time management, organizational skills, and overall readiness for autonomy
Redirect discussions to keep timely rounds
Introduce questions to facilitate discussions

Institutional Review Board approval was obtained to survey the residents as part of the Back to Bedside initiative from the University at Buffalo (approval number: STUDY00002026).

## Results

Anonymous evaluations of the faculty author were collected from 2009-2022. These included 315 total student evaluations and 620 intern and resident evaluations. Because this method had not been used by other faculty at the time of the intervention, there is no comparative data to other faculty evaluations. Furthermore, the comments were not solicited, these were voluntary end-of-rotation evaluations where learners evaluated the overall performance of the author as an educator using the Med hub/E-value online anonymous evaluations platform.

Data and comments were analyzed using constant comparative methods and were collected until reaching saturation. In accordance with grounded theory methodology, we developed codes using an iterative approach and identified three major themes described below.

1. Created an enabling environment: (a) “encouraged discussions and engagement from the entire team”; (b) “The Attending of the Day helps keep you engaged and gets you thinking critically about the patients”; (c) “the attending of the day really encouraged involvement from the whole team including students”; (d) “Stimulated excellent group discussions and prompted critical thinking”; and (e) “the technique helped inspire critical thinking and learning.”

2. Provided autonomy: (a) “the teaching philosophy allowed autonomy in decision making”; (b) “allowed for a great deal of independence in management”; (c) “Not only is enjoyable but it teaches a sense of responsibility and allows us to take ownership of our actions while still have a safety net beneath us, allowed the perfect amount of autonomy”; (d) “It allowed a good perspective and gave insight to what it means to be in charge of your decisions”; (e) “The Attending of the Day ( The Pretending) forces you to engage in decision making on each patient and makes you realize your weaknesses.”

3. Improved Confidence: (a) “challenged us to become more confident in our decisions”; (b) “Fosters independence and confidence to make your own decisions”; (c) “encouraged leadership and independent decision making”; (d) “The attending of the day not only facilitated learning but also helped improve our confidence and grow more independent”; (e) “It gives more confidence managing patients and taking tough decisions.”

Based on this analysis and during the same period in 2018, a resident-led initiative received funding from the Accreditation Council for Graduate Medical Education (ACGME) Back to Bedside Grant to support the development of innovative ideas, improve physician well-being, and foster a sense of meaning in work. The “Attending of the Day” teaching method was described as an intervention to be used program-wide to improve residents’ sense of autonomy. In 2021, the Jacobs School of Medicine launched the Moments of Excellence in Education: Recognition and Inspiration (MEE:RI) program that gives students a way to recognize exemplary moments of teaching they encounter. The “Attending of the Day” experiential technique received recognition as a transformative experience in the students’ medical education. All of the above confirms the impact that the “Attending of the Day” model has had on learners both in undergraduate education (UME) as well as graduate medical education (GME) and has encouraged more faculty to adapt it during their inpatient rounds.

## Discussion

The evaluation of learner autonomy brings several advantages. For the learner, it brings reflection on and an awareness of one’s own competencies and can contribute to improving and regulating their learning process. For the advisor, it helps them identify the strengths and weaknesses of the learner as well as areas in which support is needed [8]. Goldsmith et al. describe four supervisory styles ranging from a minimalist philosophy to a preference for high involvement in direct patient care [9]. The “Attending of the Day” can be used by supervisors following any style.

Kolb’s experiential learning theory is a holistic perspective that combines experience, perception, cognition, and behavior. Building upon earlier work by John Dewey and Kurt Levin, American educational theorist David A. Kolb believes “learning is the process whereby knowledge is created through the transformation of experience” [10]. The theory presents a cyclical model of learning, consisting of four stages.

The decision to trust a medical trainee with the critical responsibility to care for a patient is fundamental to clinical training. When carefully and deliberately made, such decisions serve as significant stimuli for learning and shape the assessment of trainees. Holding back entrustment decisions too much may hamper the trainee’s development toward unsupervised practice. When carelessly made, however, they jeopardize patient safety [2].

The limitation to this study is that the technique has only been used by one individual (it is currently being used by other faculty members with some variations). This method evaluates level 1 of Kirkpatrick’s model: learner reaction to the intervention. As previously stated, given the positive reviews that this innovation has received, the “Attending of the Day” was implemented as part of the residency program’s initiative in the setting of the ACGME’s Back to Bedside. Two more faculty have started implementing this technique during their inpatient rounds (both experienced it during their training at our institution).

## Conclusions

Structural changes in medical education have eroded learner autonomy. Autonomy is a key element of experiential learning; some might believe that supervision and autonomy are diametrically opposed. Literature has shown, however, that educators can both effectively supervise and still grant autonomy to learners. 

The “Attending of the Day” or “The Pretending” is an intervention that standardizes an approach to achieving this balance by creating a secure environment for both supervisors and trainees to build that trust and assess areas for improvement without compromising residents’ education or patient safety. It is more likely to result in more competent and confident physicians as a result of that progressive entrustment. Achieving one of Daniel Pink’s trio: the Autonomy-Mastery-Purpose will bring our residents one step closer to motivating them to work harder and perform better.
